# Delayed hypersensitivity reaction to cosmetic filler following two COVID-19 vaccinations and infection

**DOI:** 10.1186/s13223-023-00788-1

**Published:** 2023-04-20

**Authors:** Safaa Azzouz, Derek Lanoue, Katéri Champagne, Genevieve Genest

**Affiliations:** 1 Division of Internal Medicine, McGill University Health Centre, Montréal, Québec Canada; 2 Division of Allergy and Immunology, McGill University Health Centre, Montréal, Québec Canada; 3 Institut de Médecine du Sommeil, Town of Mount Royal, QC Canada

**Keywords:** Hyaluronic acid injection, Cosmetic filler, Delayed hypersensitivity reactions, COVID vaccine

## Abstract

**Background:**

With ongoing COVID-19 vaccination schedules and the popularity of cosmetic fillers, it is important to examine and record associated adverse reactions to a more general audience of health care professionals. Case reports exist in subspecialty journals outlining reactions after SARS-CoV-2 infection and vaccination. This is one of the first cases published in Canada, and it highlights priorities and challenges faced by physicians in assessing and managing patients presenting with adverse reactions post vaccination.

**Case Presentation:**

We present a case of a 43 -year-old women with delayed type 4 hypersensitivity reaction to hyaluronic acid cosmetic filler triggered by COVID-19 mRNA vaccination. We outline the clinical presentation, diagnosis, complications, and treatment of a late inflammatory reaction to hyaluronic acid filler and highlight the treatment priorities for clinicians faced with similar presentations.

**Conclusion:**

The differential diagnosis of delayed onset nodules formation post filler injection is broad and includes redistribution of fillers, inflammatory reaction to biofilm, and delayed hypersensitivity reaction. As result, in order to make the right diagnosis, administer the appropriate treatment and achieve great cosmetic results, we highly recommend seeking expert opinion from dermatologist, plastic surgeon and allergist immunologist in a timely manner.

## Background

Hyaluronic acid is a natural component of the dermis which has become the dominant cosmetic filler due to its favourable safety profile. However, adverse reactions have been reported, most commonly late inflammatory reactions. Infections and vaccinations have been proposed as potential triggers for late inflammatory reactions (LIRs). Case reports exist in subspecialty journals outlining reactions after SARS-CoV-2 infection and vaccination. We outline the clinical presentation, diagnosis, complications, and treatment of a late inflammatory reaction to hyaluronic acid filler and discuss some treatment challenges faced by clinicians with similar presentations.

## Case

**Fig. 1:**
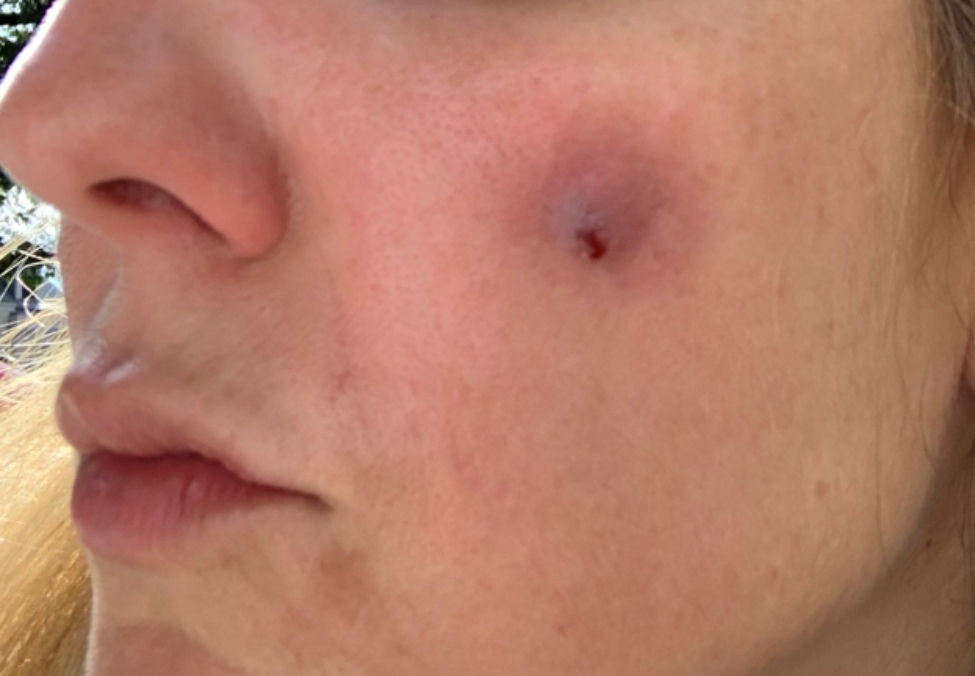
Raised inflamed erythematous papule involving the left cheek

**Fig. 2:**
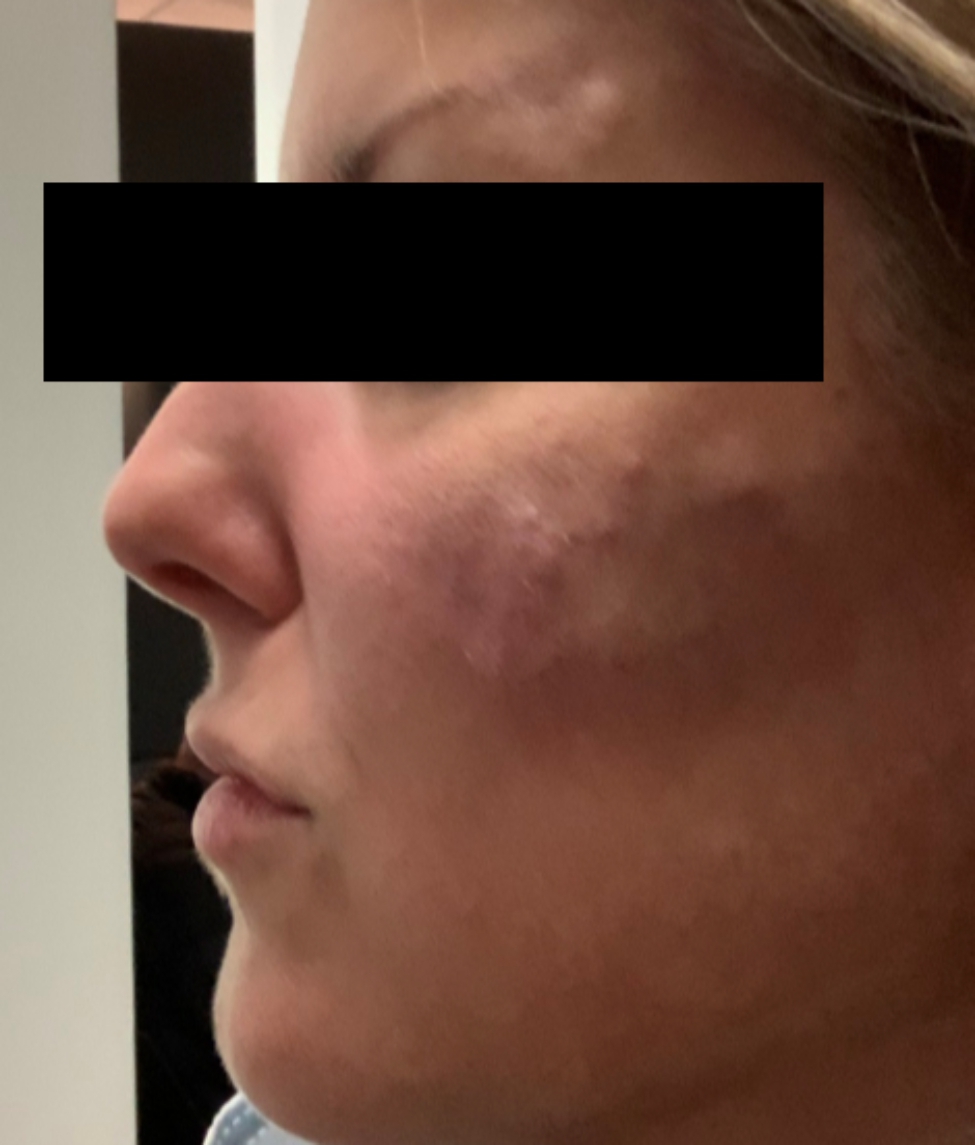
Multiples areas of induration in keeping with delayed onset nodules

**Fig. 3:**
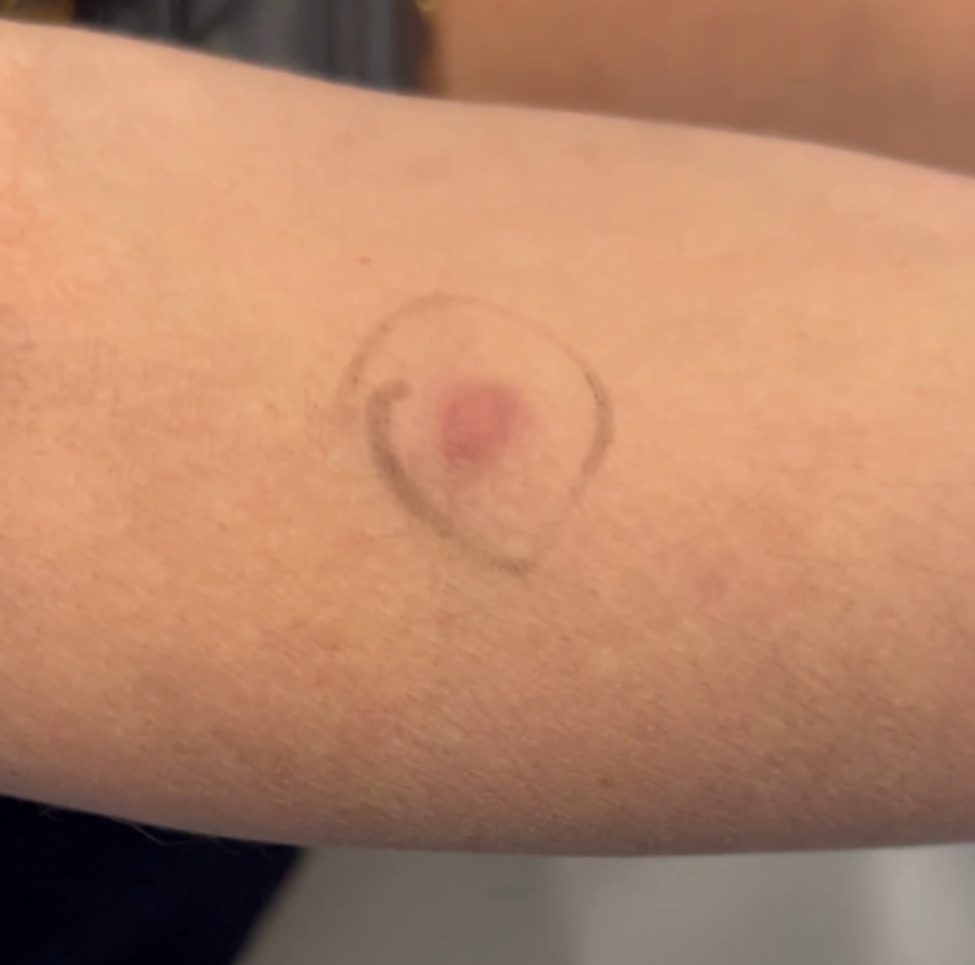
Positive delayed read intradermal skin testing to Volux /Volbella with lidocaine HA products by Juvederm

A 43-year-old woman known for a hereditary connective tissue disorder (HCTD), Hashimoto’s thyroiditis and craniopharyngioma, presented with hard subcutaneous nodules in her face. She had been previously well, receiving multiple injections of hyaluronic acid (HA, Juvéderm ® Volux and Volbella with Lidocaine, Allergan) to treat her redundant skin folds from her HCTD without adverse reactions for the last 3 years. Other cosmetic procedures include reconstructive saline breast implants which was complicated by a severe invasive Streptococcus A infection two years ago. She received her first dose of the Moderna COVID-19 vaccine in April 2021. Three weeks later, she underwent routine facial injections with HA. The following day, she developed a large erythematous pustule involving the left cheek at the site of HA injection (Fig. 1), which persisted despite multiple courses of antibiotics (cefadroxil, cephalexin and doxycycline). In July 2021, she received her second dose of COVID-19 Moderna Vaccine. Within 24 h, she developed profound malaise and facial edema, unresponsive to intravenous antibiotics or epinephrine. In the following weeks, new nodules and indurations erupted on her cheeks and chin, at the sites of previous HA injections (Fig. 2), later confirmed by ultrasound and magnetic resonance imaging (MRI). The differential diagnosis of delayed onset nodules is broad and includes redistribution of fillers, inflammatory reaction to biofilm, and delayed hypersensitivity reaction to the filler or Autoimmune Syndrome Induced by Adjuvant (ASIA). To determine the etiology of these nodules, a biopsy was performed which showed a non-specific perivascular polymorphous infiltrate with dermal fibrodysplasia without evidence of granuloma formation, infection, sarcoidosis or lupus erythematosus tumidus. Extensive laboratory workup was benign and included normal complement 3 and 4 levels, normal C1 inhibitor level and function, negative anti-nuclear and extractable nuclear antibodies. At this point, delayed hypersensitivity reaction to dermal fillers was confirmed by positive delayed read intradermal testing to Volux /Volbella→ with lidocaine HA products by JuvedermⓇ (Fig. 3); COVID-19 vaccination was thought to have triggered her reaction because of symptom onset with both doses of vaccine. From a treatment perspective, the patient was started on oral prednisone which led to visible nodule regression. Unfortunately, prednisone was not tolerated due to important psychomotor side effects. She subsequently received local steroid injections by a dermatologist and later, hyaluronidase injections by a plastic surgeon with moderate symptomatic improvement and nodule regression. To achieve cosmetic repair from ongoing residual scarring (after failing Botox, and platelet rich plasma), the patient continued HA injections and subsequently developed COVID-19 infection which led to the development of multiple new subcutaneous nodules. She was prescribed perindopril and colchicine for management of acute disease. Unfortunately, she couldn’t tolerate the ACE inhibitor and did not show clinical improvement with colchicine. An autologous fat transplant also failed to improve her appearance. She is currently avoiding further HA injections but remains permanently disfigured from her HA reactions.

## Discussion

Hyaluronic acid is a frequently used cosmetic filler. It is a natural polysaccharide that constitutes the dermis and helps keep the moisture of the skin by water retention. Intact, HA is not usually considered to be immunogenic. However, HA is metabolised to low molecular weight HA (LMW-HA) after 3–5 months; both LMW-HA and excipients used to stabilise HA in solution can be immunogenic and trigger hypersensitivity reactions in up to 0.8-1.5% of patients across various brands [1].

In our patient, we suspected a delayed inflammatory reaction to HA based upon nodules developing at previous sites of HA injections, but this is a diagnosis of exclusion. After ruling out infection, granuloma formation, Lupus and sarcoidosis we looked to confirm HA sensitization. To do so, intradermal testing (IDT) with 0.1ml of suspect HA filler was performed as previously described in the literature and read 3–4 weeks after application [2]. This yielded an inflammatory nodule at the site of IDT within 2 weeks of application, consistent with a delayed inflammatory reaction to HA (Fig. 3); IDT with saline was negative.

Delayed Type IV hypersensitivity/delayed inflammatory reactions usually manifest as induration, edema, nodules, and granuloma at the injection site [3, 1]. The mechanism of Type IV/delayed inflammatory reaction remains to be elucidated, but one theory is that both LMW-HA and HA oligosaccharides can haptenize self-protein and render them immunogenic. These haptenized proteins are taken up by dendritic cells and presented to naïve T cells, initiating an adaptive T-cell response. This leads to proinflammatory cytokine release, influx of inflammatory cells and causes tissue damage resulting in the observed local skin reaction [1]. Delayed hypersensitivity and inflammatory reactions may be triggered by an infection or vaccination (most previous reports detail Influenza or Herpes Zoster vaccine) as was the case for our patient; likely through vaccine-induced pro-inflammatory cytokine production required to prime an adaptive T cell response [4, 5]. We speculate that the COVID vaccine provided the necessary pro-inflammatory stimulus to amplify the adaptive T-cell response from the LMW-HA in our patient.

We observed a relapse of the patient’s symptoms after natural COVID-19 infection in the presence of continuous HA exposure. Similar to the COVID-19 vaccine, COVID-19 infection may have provided the necessary inflammatory stimulus to re-activate an existing memory T cell response to LMW-HA. Alternatively, COVID-19 spike protein binds the transmembrane protein angiotensin converting enzyme 2 (ACE2) for viral entry. This neutralizes ACE2’s physiologic activity in the dermis which is to convert angiotensin I and II into angiotensin 1–7 that bind Mas receptors and exhibit anti-inflammatory properties; ACE2 bound to COVID 19 spike protein causes internalization and degradation of ACE2 which reduces its bioavailability. Angiotensin is thus preferentially metabolized by ACE into angiotensin II. Angiotensin II binds angiotensin I and II receptors, and depending which receptor is most solicited, this can lead to enhanced inflammation and fibrosis. This mechanism may provide an additional inflammatory environment required for a Th1/CD8 T-cell response [6].

According to published reports available, initial management of late inflammatory reactions (LIRs) to HA should be with antibiotics that have known anti-inflammatory actions (doxycycline or tetracycline). In patients who do not respond to antibiotics, intralesional steroid and hyaluronidase injections have been used. Unfortunately, both are associated with cosmetic side effects; steroid injections can increase local skin atrophy and hyaluronidase can results in the spread of bacterial biofilms. Delayed onset nodules should be biopsied by skin care specialists to exclude granuloma formation, infection, sarcoidosis or lupus erythematosus tumidus [7]. Resurfacing can be achieved by laser, dermabrasion, surgical resection, or radial sound shockwave technology [8].

Throughout the COVID-19 pandemic, there have been reports of delayed immune reactions at the site of HA-injection following COVID-19 vaccination including 3 cases from the Moderna Phase III trial, reported by the FDA, all patients achieving complete resolution over time [9]. Given the increased recognition of this condition, a review of 13 cases was published, all in women who had sustained reactions to cosmetic filler following COVID-19 vaccination. Six patients reacted to the mRNA Pfizer vaccine and seven reacted to the mRNA Moderna vaccine. Most patients reacted within 1–10 days after vaccination and did not respond to antihistamines. Effective therapies include HA avoidance which leads to symptom improvement over several weeks to months, systemic steroids, and hyaluronidase injections. Interestingly, angiotensin converting enzyme inhibition (ACE-i) with Lisinopril was also successfully trialed and effective likely through reducing the production of angiotensin II and its pro-inflammatory downstream effects [6].

In our patient, systemic steroids and intralesional steroids/HA were either poorly tolerated or ineffective. Despite the lack of granuloma formation on biopsy, we trialed ACE-i for lack of treatment options. Unfortunately, ACE-i was also poorly tolerated. While colchicine has been proposed as a treatment to inhibit pro-inflammatory cytokine secretion, this was also ineffective in our patient. We finally recommended strict HA avoidance and while this has halted new nodule appearance and led to nodule regression, she remains permanently disfigured from her HA reactions. She continues to be managed by plastic surgery who are considering autologous fat infusions to improve cosmetic appearance.

## Conclusion

Given the increasing use of dermal fillers and prevalence of COVID-19 vaccine use as well as the spread of COVID-19 infection, general practitioners and specialists alike should be aware of the delayed hypersensitivity reactions that may occur at the site of hyaluronic acid injections.

We presented the case of a patient who developed a confirmed delayed hypersensitivity reaction to HA triggered by the COVID-19 vaccine and later by COVID-19 infection. Symptoms persisted for over 1.5 years while in most published cases, vaccine or virus-provoked delayed hypersensitivity/inflammatory reactions to HA are self-limited and respond favourably to therapy. This is likely due to continued HA injections leading to ongoing inflammation, permanent skin damage and exacerbated by a ubiquitous viral infection. Cosmetic procedures are not always readily reported to physicians by the patient, but they can sometimes have serious adverse effects. This case highlights the importance of early recognition of dermal filler reactions as well as culprit avoidance to prevent permanent sequela.

## Data Availability

N/A.

## List of abbreviations

HCTD: Hereditary Connective Tissue Disorder
ACE-i: Angiotensin Converting Enzyme inhibitor
HA: Hyaluronic Acid
MRI: Magnetic Resonance Imaging
ASIA: Autoimmune Syndrome Induced by Adjuvant
LMW-HA: Low Molecular Weight HA
IDT: Intradermal Testing
ACE2: Angiotensin Converting Enzyme 2
LIRs: Late Inflammatory Reactions
